# Theory of Worms

**Published:** 1811-10

**Authors:** W. Hamilton

**Affiliations:** Surgeon. Ipswich


*To the Editors of the Medical and Physical Journal.*

### *Theory of Worms.*

Gentlemen,

On lately perusing the American Transactions, my attention was attracted by a curious history of a living snake in a living horse's eye, related to Dr. Morgan, professor of physic in Philadelphia. The same story is related in the same volume by Mr. Hopkinson, with this difference only, that the latter calls it a *worm*. That living animals, especially worms, have been discovered in almost every organ of the human frame, is a fact as well established as any in physiology. The numerous cases related by our best authors, from the time of Hippocrates down to the present, sufficiently warrant this conclusion. Our ancestors having left us in possession of the facts, it therefore becomes our duty to arrange and digest them in such a manner, as to elicit their explanation.

Those who have attempted this may be divided into two classes, viz. those who have supported the doctrine of equivocal generation, and those who argue for the production of every animal from an egg furnished by the female and fecundated by the male. Harvey stands distinguished as being among the first supporters of this doctrine, which is now so well well established as to require no further explanation, the former being justly exploded.

The ovarian doctrine being therefore admitted, I adopt the premises, and proceed to the explanation of the phenomena of worms being found in the different organs of animals. Their most common abode is in the stomach and intestines, but they are also found in almost every organ, as the following cases prove. The history of a jointed worm near twenty inches long and three broad, found in the liver of Mrs. Holt, related in the Essays of the Society of Physicians of London. A similar case is published in the second volume of the Edinburgh Medical Essays. The celebrated Ruysch has related many cases of worms found in the lungs, kidneys, arteries, liver, cystic duct, biliary pores, gall bladder, and even in the brain itself. Old authors have distinguished them by the names of the organs in which they have been found, as dentales, gingivales, pulmonarii, cardiaci, sanguinarii, cutanei, umbilicales, hepatici salivales, &c. &c.

It being already granted that these insects originate in eggs, I shall now endeavour to point out the manner in which they arrive at the respective organs in which they have been found.

There are only two ways by which they can arrive at the respective organs mentioned above. The one by or through the skin, the other by the ova being received into the stomach with the food, and from thence proceed through the medium of the circulation (as explained below) to the respective organs in which they are discovered. The guinea worm forms an example of the first, it being generally found in the legs, where the parent deposits its egg. This is sufficiently corroborated by Linnæus's history of his own case.

It is impossible, however, for the ova of insects to pass in this manner into the viscera contained within the abdomen or thorax, we must therefore have recourse to the circulation.

I therefore suppose these ova are received with the food into the stomach, and in proportion to their minuteness pass either through the lacteals with the chyle, or into the lower intestine with the fœces. Those with the chyle enter the circulation at the thoracic duct, and these remaining in the intestine, generally find a nidus in the villous coat or in the duplicatures of the colon, where they may not only arrive at maturity, but also greatly augment their number, each being endowed with male and female organs.* This may account for their being found more frequently in the intestines than any of the other viscera. Passing thus with the circu-

* Viz. Teres, tenia, and lumbricus, possess this power.

lation lation these ova are deposited by the blood in some appropriate organ, where they receive that degree of warmth and nutrition necessary to call forth their organic existence. According to this theory the produce of these ova are liable to be found in every part of the human frame, especially in the abdominal viscera, which receive the blood more immediately from the center of circulation and in larger proportion, owing to the functions those organs have to perform, as the liver, kidneys, &c. &c. How these ova escape digestion on being received into the stomach shall now be examined. The best experiments and observations we possess on this subject are those of the celebrated John Hunter. He found several substances insoluble in the gastric juice, as horn, husk, shells of all ova, and the essential oils of animals and vegetables. He says, "these oils, although indigestible, are miscible or soluble in the gastric juice or chyle, by which means they become medicinal from their stimulating power." A little farther on he says, "the essential oils of vegetables, but more particularly that of animals, would seem to pervade the very substance of those animals whose food contains much of this oil; thus we find sea birds, whose constant food is fish, taste very strongly of fish."

Mr. Hunter mentions also fluids as being difficult of digestion from the minuteness of their particles and want of solidity. We have, therefore, only to suppose that the size of the ova, which pass with the chyle, are equal in minuteness to the particles of a fluid or the oils above noticed, which was found to have pervaded every part of the system. Those who are at all conversant with the history of insects will readily grant this supposition.

The fact that the greater number of worms are discovered in the intestines, sufficiently indicates that few ova only are of such a degree of minuteness as to be absorbed by the lacteals. Those larger passing into the intestines escape digestion, either from the gastric juice having no action on their horny shells, or their being possessed of vitality. In this manner we daily see the skins and husks of various kind of fruits pass off with the fœces without undergoing the least alteration.

The ova which pass into the intestine with the fœces are either nurtured in the duplicatures of the colon, or expelled by the rectum. I mentioned that should any of these insects be possessed of vitality, that alone prevented their digestion. This I conceive to be sufficiently proved from the dissolution of the stomach itself, by the gastric juice after death, and more especially by the large living worms found in this viscus. The objection to this, that we are daily swallowing living oysters which are soon digested, is of no weight, as

 these these oysters must soon die, being lacerated and deprived of their shell, when digestion will of course follow.

Ruysch says, "I have had reason to doubt whether, as Harvey and his followers affirm, all animals are produced out of eggs, from worms being found in the arteries of living horses, and in the parenchymatous or glandular substance of the liver, &c." Neither analogy nor the invariable laws of nature can lead us to suppose these organs endowed with a self generating power, capable of producing new species of animals without parents. We must therefore adopt the above doctrine, which, without straining facts, satisfactorily explains this phenomenon, as we can scarcely suppose that the parent can gain access to deposit her eggs in these internal organs. It may be asked why young people are more subject to worms than adults? This is true, only so far as relates to intestinal worms, the increase of which, at this period, I have heard attributed to the greater succulency of our juices. It is however more probably owing to the larger quantity of food, especially vegetable, which is consumed about this time.

Vallisnieri has sufficiently proved that the intestinal worms propagate their species in the intestine. from which he concludes that they are generated there, and not received into the circulation with the food, &c. This doubtless accounts for their great increase, but does not explain the production of the parent. Worms have not only been found in infants but even in fœtus's. This, in my opinion, is an incontestable proof of their being received into the circulation of the mother, and from thence proceed through the same medium to the fœtus.

Several cases of worms being voided with the urine have lately been published. This is another strong proof of the present theory; it being well known that a very large quantity of blood circulates through the kidneys with remarkable celerity.

Having thus shewn that the eggs of insects not only enter the stomach and alimentary canal, but are also absorbed undigested by the lacteals, after which they must enter the circulation with the nutrition of the food, and be deposited with the same in some appropriate organ; the explanation of worms being discovered in every part of the animal frame becomes equally easy, all parts being duly supplied with blood.

W. HAMILTON, Surgeon.

*Ipswich, July* 26, 1811.

